# Beyond time spent online: exploring functional motives, discourse-related self-appraisals, and social media addiction-like symptoms

**DOI:** 10.3389/fpsyg.2026.1875894

**Published:** 2026-10-27

**Authors:** Mario Lehenbauer-Baum, Armin Klaps, Zuzana Kovacovsky, Birgit Ursula Stetina

**Affiliations:** Department of Clinical Psychology, Faculty of Psychology, Sigmund Freud Private University, Vienna, Austria

**Keywords:** behavioral addiction, escapism, social media, social media addiction, usage motives

## Abstract

**Introduction:**

Research has increasingly examined excessive social media (SM) use in relation to negative mental health outcomes, often conceptualized as SM “addiction” or “problematic use”, and assessed using self-report scales such as the Bergen Social Media Addiction Scale (BSMAS). While the BSMAS assesses self-reported addiction-like symptoms, scores may be associated not only with time spent using SM, but also with the functions SM serves and discourse-related self-appraisals through which individuals evaluate their own use. This exploratory pilot study examined these dimensions.

**Methods:**

We developed two exploratory instruments assessing functional motives and discourse-related self-appraisals. A convenience sample of 291 SM users completed the BSMAS, SM use questions, and both instruments. Exploratory factor analyses, correlations and hierarchical regressions examined associations with BSMAS scores. Three sensitivity analyses examined potential conceptual overlap with BSMAS item content.

**Results:**

The Functional SM Use Survey yielded five domains: emotional self-regulatory engagement and escapism, global wellbeing benefit, entertainment and stimulation-driven engagement, instrumental and goal-directed use, and social validation and self-presentation. The Discourse-Based Survey yielded three domains: socially driven self-judgment, warning-triggered self-appraisal, and perceived over-pathologization and normative dissent. Weekly SM use was significantly associated with BSMAS scores in Step 1 but no longer significant in the fully adjusted model. Emotional self-regulatory engagement and escapism (β = 0.438) and warning-triggered self-appraisal (β = 0.273) remained statistically significant; the model explained 49.8% of the variance. The socially driven self-judgment association weakened after removing overlapping items, whereas the warning-triggered self-appraisal association changed minimally after removal of the BSMAS work/study-impact item. This does not establish conceptual independence between warning-triggered self-appraisal and perceived problematic use. Removing the BSMAS mood-modification item attenuated, but did not eliminate, the emotional self-regulatory engagement and escapism association.

**Discussion:**

BSMAS scores were associated with reported SM use, coping-oriented motives and discourse-related self-appraisals. Sensitivity findings indicate that some associations were attenuated after removing overlapping evaluative content, whereas others changed minimally after targeted item removal. These analyses do not establish conceptual independence among the measures. These exploratory findings indicate associations between self-reported addiction-like symptoms, SM motives and discourse-related self-appraisals, but the conceptual distinctiveness of the newly proposed domains requires independent validation.

## 1 Introduction

For a large group of people, the use of social media (SM) platforms is deeply embedded in everyday life. These platforms provide multiple forms of entertainment and opportunities for self-representation; on the same SM platform, users can satisfy multiple needs, such as social interaction, entertainment, passing time, and information seeking (Quan-Haase and Young, 2010; Whiting and Williams, 2013). Emerging research also suggests that SM use may function as a coping strategy, with individuals turning to SM to dampen negative affect or enhance positive mood (Brown et al., 2026; Yildiz and Uslu, 2023). These findings suggest that SM use can serve multiple psychological functions, including goal-directed, affect-regulatory, and hedonic purposes, with individuals engaging with the same platform for different psychological reasons.

Against this background of various functional motives, public and scientific debates have increasingly focused on concerns about SM use, even though findings regarding its associations with mental health remain mixed. Some studies suggest only small to moderate links (Jiao et al., 2025; Keles et al., 2020; Shannon et al., 2022), and these inconsistent findings may partly be related to differences in motives for SM use and psychological characteristics such as emotion-regulation skills. For example, Thorell et al. (2024) found that motives such as escapism, as well as using SM to form new friendships among individuals with poorer social skills, were associated with higher self-reported addiction-like symptoms. In contrast, Gu et al. (2022) found that emotion regulation skills like mindfulness were associated with better mental health outcomes among SM users; mindfulness was associated with decreased distress tied to social comparisons on SM and reduced worry about social media posts.

Furthermore, excessive SM use is often labeled as “problematic” or “addictive” in the scientific literature (Kuss and Griffiths, 2011). It is therefore not surprising that concepts such as “social media addiction”, “too much screen time”, and “digital wellbeing” have become popular not only in scientific research, but also in public discourse, media reporting, and everyday conversations. These public narratives may provide interpretive frameworks through which individuals evaluate their own media use. Although problematic or addictive SM use is commonly operationalized using self-report questionnaires assessing addiction-like symptoms, individuals' responses may reflect not only their patterns of media use, but also how they interpret and evaluate these behaviors in relation to broader public narratives. Accordingly, the present study does not focus on determining whether individuals meet criteria for clinical addiction, but on understanding how functional usage motives and discourse-related self-appraisals are associated with self-reported addiction-like symptoms as measured by the Bergen Social Media Addiction Scale (BSMAS).

### 1.1 Behavioral addiction models of social media use

Concerns about new technologies taking over everyday life are not new; “TV addiction” was already discussed as a pathological construct as early as 1991 (McIlwraith et al., 1991), and measures were developed for this novel construct (Horvath, 2004). Around the same time, the idea of “non-chemical addictions” emerged (Marks, 1990), followed by increasing research on behavioral addictions and, ultimately, the inclusion of Internet Gaming Disorder in DSM-5 Section III in 2013 (American Psychiatric Association, 2013). Since then, the concept of behavioral addictions in the context of digital media has received growing empirical attention, with studies linking elevated symptom scores to a range of adverse psychological outcomes. At the same time, however, their conceptualization, diagnostic boundaries, and applicability to everyday digital behaviors have remained the subject of considerable scientific debate.

Conceptually, criticism has emerged regarding the application of substance-use criteria to everyday activities (Billieux et al., 2015; Kardefelt-Winther et al., 2017), and “technological addiction” as a construct has been critically examined early on (Griffiths, 2000). Authors also criticize inconsistent classification criteria for behavioral addiction, limited evidence regarding etiology and course (Petry and O'Brien, 2013), and a lack of clarity between adaptive (or engaged) and maladaptive (or addicted) use of digital media (Lehenbauer-Baum et al., 2015; Lehenbauer-Baum and Fohringer, 2015). Furthermore, “behavioral addictions” represent a highly heterogeneous group that has included hypersexuality, exercise addiction, muscle dysmorphic disorder, compulsive buying, and workaholism, among others (Demetrovics and Griffiths, 2012).

In the context of SM addiction, screening tools have frequently been built around established addiction criteria, followed by investigations of whether familiar addiction-related risk factors are also present, an approach that has been criticized as potentially confirmatory rather than theory-generating (Demetrovics and Griffiths, 2012). Some authors argue that this approach may risk over-pathologizing by turning ordinary coping, leisure, and social connection behaviors into symptoms of contemporary pathologies, raising concerns about where to draw the line between adaptive behavior and clinically meaningful impairment (Mihordin, 2012). At the same time, proponents of SM addiction argue that, for subgroups of individuals, excessive engagement can indeed become associated with significant impairment and therefore warrants clinical attention.

Following a similar approach, SM addiction has been proposed as a pathological construct, and instruments such as the Bergen Social Media Addiction Scale (BSMAS), itself derived from the Bergen Facebook Addiction Scale (Andreassen et al., 2012), were developed to operationalize addiction-like symptoms associated with SM use using “classical” addiction criteria such as salience, mood modification, tolerance, withdrawal, conflict, and relapse (Zarate et al., 2023). Numerous studies have reported associations between elevated BSMAS scores and constructs like depression (Santini et al., 2025; Višnjić et al., 2023), anxiety (Peng and Liao, 2023; Kosola et al., 2024), and lower quality of life (Demirkol et al., 2025; Duradoni et al., 2020). While these findings demonstrate associations between elevated BSMAS scores and broader psychological difficulties, it is important to note that the BSMAS is a self-report instrument assessing individuals' endorsement of addiction-like symptoms rather than establishing the presence or absence of a clinical disorder. Consequently, the present study examines variation in self-reported BSMAS scores rather than social media addiction.

While some individuals experience significant impairment associated with excessive SM use, many users frequently engage with these platforms without experiencing clinically significant impairment or substantial negative consequences. This raises an important question: If some users view similar levels of engagement as adaptive while others view them as problematic, what shapes these different evaluations? Beyond actual usage behavior, individuals may differ not only in the psychological functions that SM serves, but also in the broader social and cultural narratives through which they evaluate their own behavior. Because the BSMAS relies on self-reports, these functional and interpretive processes may be associated with how individuals endorse addiction-like symptoms. The latter perspective forms the basis of the present study and is examined through individuals' discourse-related self-appraisals of their own SM use.

### 1.2 Discourse-related self-appraisals

In a cultural environment where concerns about “screen time,” “digital detox,” and the potential harms of excessive SM use are widely discussed, individuals may evaluate their media behavior in relation to broader social and cultural narratives. The present study does not examine public discourse itself or individuals' exposure to such discourse. Rather, it focuses on discourse-related self-appraisals, defined here as individuals' evaluations and interpretations of their own SM use in relation to perceived social judgments, public and media narratives, expert warnings, and digital self-tracking feedback. These appraisals may therefore reflect how individuals relate their own behavior to culturally available ways of describing and evaluating SM use. Importantly, the construct refers to individuals' self-reported appraisals in relation to discourse-related cues rather than demonstrating that public discourse directly influences their behavior or self-perceptions.

One theoretical perspective relevant to such self-appraisals is Social Comparison Theory (Festinger, 1954), which proposes that individuals may evaluate themselves in relation to others and their judgments, particularly when objective standards for evaluation are unclear. Applied to SM use, the boundary between frequent, excessive, and “problematic” use may not always be readily apparent to individuals. Perceived judgments from others and socially available standards may therefore provide reference points against which individuals evaluate their own behavior. From this perspective, discourse-related self-appraisals may partly reflect how individuals relate their own SM use to external evaluative information when judging whether that use is excessive or problematic.

Self-labeling approaches offer a complementary perspective by examining how individuals recognize and categorize their experiences or behaviors using problem-related or diagnostic labels. In the mental-health literature, for example, self-labeling has been examined in terms of whether individuals recognize their own symptoms as constituting a mental-health problem, with implications for subsequent help-seeking (Thoits, 2026). Applied to SM use, socially available terms such as “social media addiction” or “problematic social media use” may similarly provide categories through which individuals consider and describe their own behavior. Importantly, individuals need not simply adopt socially available labels. Madsen and Raikhel (2025), for example, found that college students frequently encountered diagnostic mental-health content on social media but were often skeptical of its accuracy and reluctant to apply the label “self-diagnosed” to themselves, even when privately identifying with particular conditions. Their findings illustrate that individuals may both identify with and resist socially available psychological labels, depending partly on their perceived legitimacy and applicability.

Particularly relevant evidence comes from Anderson and Wood (2025), who found that Instagram users overestimated their addiction to the platform and argued that perceptions of addiction may partly arise from popular media's frequent labeling of SM as addictive rather than habit-forming. In an experimental study, framing frequent Instagram use as an addiction reduced perceived control over SM use and increased self-blame for overuse. These findings provide experimental evidence that the language used to characterize frequent SM use can affect users' perceptions of their own behavior, including their perceived control and self-blame. The present study does not test such framing effects or assume that exposure to public narratives causes self-perceptions. Rather, it examines individual differences in the extent to which perceived social judgments, addiction-related labels, warnings, and other discourse-related cues are reflected in individuals' self-appraisals of their own SM use.

Despite ongoing conceptual debates surrounding behavioral addiction, comparatively little quantitative research has examined whether such discourse-related self-appraisals are associated with individuals' reports of addiction-like SM symptoms. For example, most psychometric work on the BSMAS has focused on its internal structure, reliability, and associations with mental health outcomes, while less attention has been paid to the interpretive processes that may also be associated with individuals' responses. Accordingly, while functional motives may help explain differences in engagement, discourse-related self-appraisals may additionally be associated with how individuals interpret that engagement when evaluating themselves on questionnaires assessing addiction-like symptoms. Together, these perspectives suggest that individual differences in BSMAS scores may be associated not only with how individuals use social media, but also with how they interpret and evaluate that use.

Building on these considerations, the present exploratory pilot study examined whether variation in BSMAS scores is associated not only with the functional motives underlying SM use, but also with the extent to which individuals evaluate their own SM use in relation to contemporary public narratives surrounding addiction, screen time, and digital wellbeing. To address these objectives, we developed two novel instruments. The first assessed a range of psychological functions of SM use, including emotional self-regulation, entertainment and stimulation, social validation, and instrumental and goal-directed use. The second assessed discourse-related self-appraisals, capturing individuals' evaluations of their own SM use in relation to perceived social judgments, public narratives surrounding social media addiction and digital excess, expert warnings, and digital usage feedback. Beyond time spent on social media, the present study examined whether functional motives and discourse-related self-appraisals are associated with individual differences in BSMAS scores.

## 2 Materials and methods

### 2.1 Instruments

To examine functional motives for SM use and discourse-related self-appraisals of individuals' own SM use, and how these dimensions relate to self-reported addiction-like symptoms, the following instruments were used:

#### 2.1.1 Bergen social media addiction scale (BSMAS)

The BSMAS assesses self-reported addiction-like symptoms associated with SM use, using six items reflecting the behavioral addiction components of salience, mood modification, tolerance, withdrawal, conflict, and relapse (Andreassen et al., 2012, 2016). Items were rated on a 5-point Likert scale ranging from 1 (very rarely) to 5 (very often) and were summed to yield a total score ranging from 6 to 30, with higher scores indicating greater endorsement of addiction-like symptoms. Internal consistency in the present sample was α = 0.78. As the BSMAS is a self-report instrument, it was used in the present study for participants' self-reported addiction-like symptoms rather than to infer a clinical diagnosis.

#### 2.1.2 Weekly SM use

Participants reported their estimated amount of SM use; responses were expressed in hours per week and used as the self-reported usage-duration indicator in the correlational and regression analyses.

#### 2.1.3 Functional SM use survey

Because no existing survey adequately captured the broad range of functional motives underlying contemporary SM use, we developed a new Functional SM Use Survey specifically for this pilot study. All newly developed items were generated and administered in English (for this Functional SM Use Survey as well as the Discourse-Based Survey); therefore, no translation or back-translation procedure was required. Item development followed a theory-driven process informed by the uses-and-gratifications framework (e.g., social interaction and information seeking), research on digital emotion regulation, and previous findings on adaptive or beneficial SM use (e.g., using SM for information, inspiration, and wellbeing support). Five licensed clinical psychologists were asked to generate potential reasons why individuals use social media, primarily formulated as first-person statements (“I use social media to…”), resulting in an initial pool of 46 items. The first author reviewed these items for clarity, conceptual relevance, comprehensiveness of motivational domains, and redundancy. Following this review, 15 items were removed, resulting in a 31-item survey administered in the present study. As this represents the first application of this instrument, the scale should be regarded as preliminary and exploratory. All items were answered on a 7-point Likert scale ranging from 1 (strongly disagree) to 7 (strongly agree). We then used exploratory factor analysis to identify the underlying factor structure and reduce the item pool (see Results).

#### 2.1.4 Discourse-based survey

Similarly, because no suitable measure of discourse-related self-evaluation of SM use was available, we developed a second instrument for this pilot study. The Discourse-Based Survey was developed to assess discourse-related self-appraisals, defined as individuals' evaluations and interpretations of their own SM use in relation to perceived social judgments, public and media narratives, expert warnings, and digital self-tracking feedback. Five licensed clinical psychologists were asked to provide items concerning behavioral addictions, over-pathologizing of everyday behavior, sociocultural perspectives on psychological labels, and public discourse surrounding digital technologies, resulting in an initial pool of 37 statements. The first author reviewed these items for conceptual relevance, clarity, and redundancy. Following this review, 10 items were removed, resulting in a 27-item survey administered in the present study. Like the Functional SM Use Survey above, this measure is novel and should be considered exploratory until replicated and validated in independent samples. All items were answered using the same 7-point Likert scale ranging from 1 (strongly disagree) to 7 (strongly agree) and were subsequently subjected to exploratory factor analysis to identify latent discourse domains (see Results).

### 2.2 Participants and procedure

The study and analysis plan were not preregistered; accordingly, all analyses should be regarded as exploratory. Following approval by the Sigmund Freud Private University Ethics Commission of the Faculties of Psychotherapy Science, Psychology and Law (BDP9FTBACO8ESA92068), participants were recruited online via Instagram and Facebook between December 1, 2025, and February 14, 2026, using SoSciSurvey. Recruitment employed a convenience sampling strategy, and participation was voluntary and uncompensated. Eligibility criteria included being at least 18 years of age and using SM for at least 1 h per week. All survey materials were presented in English.

The survey consisted of four consecutive pages presented in a fixed order. The first page contained study information and the informed-consent procedure, and participants were required to provide electronic informed consent before proceeding. The second page included demographic questions on age and gender, questions regarding SM use, including weekly hours of SM use, and the Bergen Social Media Addiction Scale (BSMAS). The third page contained the Functional SM Use Survey items, and the fourth page contained the Discourse-Based Survey items. The order of these sections was not randomized.

The original survey dataset contained 332 entries. Forty-one entries were excluded prior to analysis because they contained insufficient responses across the study measures for inclusion in the planned analyses, leaving 291 analyzable cases. No missing-value imputation was performed. All 291 participants included in the analytic sample had complete data on the variables used in the primary correlational and regression analyses. The final sample included 163 female participants (56.0%), 119 male participants (40.9%), and 8 participants (2.7%) reporting another gender identity; gender information was missing for one participant (0.3%). Mean age was 29.22 years (SD = 10.94), and participants reported an average of 21.70 h of SM use per week (SD = 12.82).

Because recruitment occurred exclusively through SM platforms, the sample likely over represents individuals who are relatively active or engaged SM users. Consequently, interpret the findings as reflecting an online convenience sample rather than the general population of SM users.

### 2.3 Statistical analyses

No a priori power analysis was conducted. The study was designed as an exploratory pilot study using a convenience sample. The fully adjusted regression contained nine predictors in a sample of *N* = 291, corresponding to approximately 32 participants per predictor. Analyses were conducted using IBM SPSS Statistics (Version 30). As both questionnaires were newly developed, exploratory factor analysis (EFA) was chosen to examine their latent structures. We evaluated sampling adequacy using the Kaiser-Meyer-Olkin (KMO) statistic and Bartlett's test of sphericity. Separate EFAs were conducted for the Functional SM Use Survey (31 items) and the Discourse-Based Survey (27 items) using principal axis factoring with direct Oblimin rotation and Kaiser normalization, as correlations between latent factors were expected.

The number of retained factors was determined by considering multiple criteria, including eigenvalues, scree plots, theoretical interpretability, and conceptual coherence. Adjacent alternative factor solutions were examined for each instrument before selecting the final models. Items were iteratively removed based on weak primary loadings, low communalities, substantial cross-loadings, and/or conceptual inconsistency with the emerging factor content. Decisions regarding item removal were based on both statistical criteria and theoretical interpretability rather than solely on numerical thresholds. The retained solutions were selected as providing the best balance between statistical adequacy and meaningful psychological domains. Because factor orientation in EFA is mathematically arbitrary, factors extracted in the direction opposite to the substantive scoring of their items were sign-reoriented for presentation where necessary. This reorientation did not alter the underlying factor solutions, communalities, observed domain sum scores, or subsequent analyses. All retained items were scored from 1 (strongly disagree) to 7 (strongly agree), with higher scores indicating stronger endorsement of the respective item content. Unweighted sum scores were computed for the retained functional and discourse domains and were used in all subsequent correlational and regression analyses. Cronbach's α coefficients were calculated to examine the internal consistency of the retained domains.

Pearson correlations were used to provide initial information about associations between the examined constructs. Statistical significance was evaluated at α =0.05; given the exploratory nature of the study, *p* values were not adjusted for multiple comparisons. A primary hierarchical regression was then conducted with BSMAS sum scores as the dependent variable. Weekly SM use was entered in Step 1, the three discourse-related self-appraisal domain scores in Step 2, and the five functional domain scores in Step 3. All predictors were entered simultaneously within their respective blocks. This ordering was used to first examine associations with discourse-related self-appraisals beyond reported usage duration and then assess whether functional motives accounted for additional variance beyond both usage duration and discourse-related self-appraisals.

To address potential conceptual overlap between the discourse survey and the BSMAS, multiple sensitivity analyses were performed. First, two otherwise identical hierarchical regression models were compared. Both included weekly SM use in Step 1 and the five functional domains in Step 3. In Step 2, the first model included the original D1 and D3 scores, whereas the second included revised D1 and D3 scores after removal of three D1 items and two D3 items that explicitly referred to addiction, excessive use, or problematic consequences. Internal consistency was additionally examined for the resulting revised D1 and D3 scores. Because D2 warning-triggered self-appraisal contained only four items, it was not included in these two D1/D3 sensitivity models; potential D2—BSMAS overlap was instead addressed separately in the second sensitivity analysis described below.

Accordingly, a second sensitivity analysis addressed potential D2—BSMAS overlap from the outcome side. The BSMAS was recalculated after excluding Item 6, which assesses whether SM use has had a negative impact on work or studies and was considered the BSMAS item most conceptually proximal to D2 judgments of excessive or problematic use. We examined internal consistency for the resulting five-item BSMAS score and repeated the primary three-step hierarchical regression using this reduced score as the dependent variable while retaining the complete four-item D2 domain.

A third sensitivity analysis addressed potential conceptual overlap between F1 emotional self-regulatory engagement and escapism and the BSMAS mood-modification item. The BSMAS was recalculated after excluding Item 3 (“How often during the last year have you used social media to forget about personal problems?”), while retaining the complete F1 domain. Internal consistency was examined for the resulting five-item BSMAS score, and the primary three-step hierarchical regression was repeated using this reduced score as the dependent variable. This analysis examined whether the unique association between F1 and BSMAS scores persisted after excluding the BSMAS item most directly reflecting mood modification.

Regression diagnostics were examined for the fully adjusted primary model. Linearity and homoscedasticity were evaluated through inspection of standardized residuals plotted against standardized predicted values, while the distribution of residuals was examined using a histogram and normal P-P plot. Potentially unusual or influential observations were examined using standardized residuals and Cook's distance. Multicollinearity was evaluated using variance inflation factors (VIFs) and tolerance values.

Given the exploratory nature of the study, the same sample was used for scale development and subsequent regression analyses. Accordingly, the resulting factor structures and regression models should be regarded as preliminary and require replication and validation in independent samples.

## 3 Results

### 3.1 Functional SM use survey

A series of EFAs using principal axis factoring with direct Oblimin rotation and Kaiser normalization was conducted, beginning with the full 31-item pool. Initial inspection of the scree plot and theoretical interpretability led us to examine a five-factor structure, which was subsequently refined through iterative item removal. In the initial 31-item solution, four items were removed because of weak primary loadings or substantial cross-loadings. The subsequent 27-item solution resulted in the removal of three additional items based on weak loadings, cross-loadings, low communalities, and/or limited conceptual fit with the emerging domains. Across five further iterations, six additional items were removed because of weak or ambiguous factor representation, low communalities, cross-loadings, or conceptual inconsistency with the emerging factor content. Decisions were therefore based jointly on statistical criteria and conceptual interpretability rather than on numerical cutoffs alone. The final EFA retained 18 items. A detailed item-removal sequence and rationale for each removed item are provided in the Supplementary material.

Sampling adequacy was good (KMO = 0.835), and Bartlett's test indicated that the correlation matrix was factorable, χ^2^(153) = 2218.28, *p* < 0.001. In the final 18-item solution, primary pattern loadings ranged from 0.443 to 0.928 in absolute magnitude, and no retained item showed a secondary pattern loading with an absolute magnitude ≥0.30. Extraction communalities ranged from 0.259 to 0.826. Five eigenvalues exceeded 1 (5.597, 2.159, 1.972, 1.207, and 1.124), followed by a sixth eigenvalue of 0.749.

Factor retention was further evaluated by comparing adjacent four- and six-factor solutions using the same final 18-item pool. The forced four-factor solution combined the global wellbeing items with social-validation and self-presentation content, resulting in a broader and conceptually heterogeneous factor. The forced six-factor solution did not yield an admissible completed solution. The initial analysis did not converge within the default maximum of 25 extraction iterations. When the analysis was repeated with the maximum increased to 100 iterations, the communality of one variable exceeded 1.0 at iteration 100 and extraction was terminated. Accordingly, statistics from the unfinished six-factor solution are not interpreted. The five-factor solution therefore provided the clearest simple structure and most conceptually coherent representation of the retained items. The complete pattern matrix and factor-correlation matrix for the retained five-factor solution are provided in Supplementary Table S4, the item-removal sequence is reported in Supplementary Table S5, and factor-retention evidence comparing the four-, five-, and six-factor solutions is summarized in Supplementary Table S6.

The final five-factor solution comprised F1) emotional self-regulatory engagement and escapism (5 items; α = 0.86), F2) global wellbeing benefit (2 items; α = 0.81), F3) entertainment and stimulation-driven engagement (4 items; α = 0.80), F4) instrumental and goal-directed use (4 items; α = 0.71), and F5) social validation and self-presentation (3 items; α = 0.69). Although F2 comprised only two items, retaining it as a separate factor was supported by both statistical and theoretical considerations. In the forced four-factor solution, the two wellbeing items were combined with social-validation and self-presentation items, resulting in a broader factor that mixed global evaluations of the benefits of SM use with more specific interpersonal and self-presentational functions. In the preferred five-factor solution, the two wellbeing items instead formed a distinct and internally coherent domain. The two wellbeing items also showed a strong inter-item association (*r* = 0.686; Spearman-Brown coefficient = 0.814), supporting their internal coherence despite the factor's brevity. Accordingly, the two-item factor was retained as a brief preliminary index of perceived global wellbeing benefit, while acknowledging that replication and further item development are warranted in future validation studies.

Factor correlations in the final five-factor solution ranged from ϕ = 0.02 to ϕ = 0.44, indicating related but non-redundant domains. The strongest factor correlation was observed between F3 entertainment and stimulation-driven engagement and F4 instrumental and goal-directed use (ϕ = 0.44). Because factor orientation in EFA is mathematically arbitrary, the factor corresponding to F5 social validation and self-presentation was initially extracted in the opposite direction to the substantive scoring of its items. For interpretive consistency, F5 was reoriented for presentation so that higher factor values correspond to greater social validation and self-presentation. This sign reversal does not alter the underlying factor solution, communalities, observed domain sum scores, or subsequent analyses. Table 1 presents the retained items and primary loadings; the complete pattern matrix is provided in the Supplementary material.

**Table 1:** Functional SM use survey: factor loadings and communalities.

Factor/Item	Loading	*h* ^2^
F1: Emotional self-regulatory engagement and escapism		
1. I use social media to “escape” from stressful situations for a moment.	0.806	0.728
2. When I feel stressed, anxious, or overwhelmed, I use social media to distract myself.	0.805	0.764
3. Social media helps me cope better with difficult emotions.	0.741	0.600
4. I use social media to calm myself down internally after exhausting or emotionally stressful situations.	0.709	0.584
5. I use social media to have the feeling that I have my emotions better under control.	0.571	0.429
F2: Global wellbeing benefit		
6. I feel that using social media supports my overall wellbeing.	0.795	0.725
7. I feel that social media has a positive impact on my daily life.	0.719	0.658
F3: Entertainment and stimulation-driven engagement		
8. I use social media because certain content strongly appeals to and stimulates me.	0.928	0.826
9. I use social media because I find the content entertaining.	0.671	0.477
10. I use social media because some content is especially stimulating for me.	0.648	0.606
11. I use social media to combat boredom.	0.492	0.384
F4: Instrumental and goal-directed use		
12. I use social media to get inspiration and ideas (e.g., for fashion, home decor, work, hobbies, new recipes, etc.).	0.693	0.564
13. I use social media to get information about topics that are important for me personally, for my job, or for my education.	0.548	0.274
14. I use social media to find out what is new online.	0.528	0.479
15. I use social media to discover new hobbies or interests.	0.521	0.375
F5: Social validation and self-presentation		
16. I use social media because positive feedback or reactions (e.g., likes, matches, messages) strengthen my self-esteem.	0.836	0.725
17. I use social media to present myself (e.g., through photos, posts, or stories).	0.658	0.478
18. I use social media to meet new people (e.g., through dating apps, groups, or communities).	0.443	0.259

Extraction method = principal axis factoring; rotation method = direct Oblimin with Kaiser normalization.

Items are numbered sequentially in this table for presentation; Supplementary Table S4 retains the original administered item numbers.

### 3.2 Discourse-based survey

Exploratory factor analyses using principal axis factoring with direct Oblimin rotation and Kaiser normalization were conducted beginning with the 27-item pool. Inspection of the initial scree plot and conceptual interpretability suggested a three-factor structure, which was subsequently examined using a forced three-factor solution.

Although five initial eigenvalues exceeded 1 in the 27-item analysis (8.563, 3.148, 1.410, 1.115, and 1.012), the fourth and fifth were close to unity. Because the Kaiser criterion therefore suggested a potentially higher-dimensional solution, a forced five-factor solution was also examined. This solution recovered recognizable precursors of the subsequently retained domains but fragmented the normative-dissent content across multiple factors and yielded an essentially single-item fifth factor, indicating over-extraction. Accordingly, factor retention was not based on the Kaiser criterion alone, but on the scree pattern, simple structure, conceptual interpretability, and comparison of alternative solutions.

Five items were removed from the initial solution because of weak primary loadings, low communalities, cross-loadings or ambiguous factor representation, and/or conceptual inconsistency with the emerging domains. A second 22-item EFA led to the removal of three additional items because of weak or diffuse loadings, low communality, or substantial cross-loading. The final EFA therefore retained 19 items. The complete item-removal sequence and rationale are provided in the Supplementary material.

Sampling adequacy was high (KMO = 0.896), and Bartlett's test indicated that the correlation matrix was factorable, χ^2^(171) = 2233.79, *p* < 0.001. In the final solution, absolute primary pattern loadings ranged from 0.391 to 0.857, no retained item showed a secondary pattern loading ≥0.30, and extraction communalities ranged from 0.219 to 0.714. Three eigenvalues exceeded 1 (6.266, 2.897, and 1.258), followed by a fourth eigenvalue of 0.933. The three-factor solution accounted for 47.0% of the extracted variance.

Factor retention was further evaluated by comparing adjacent two-, three-, and four-factor solutions using the same final 19-item pool. The two-factor solution combined socially driven self-judgment and warning-triggered self-appraisal into a broader factor, thereby obscuring the distinction between general socially mediated self-judgment and appraisal specifically triggered by warnings or usage feedback. In contrast, the four-factor solution divided perceived over-pathologization and normative dissent into two strongly related subcomponents. The two D3-derived subcomponents were correlated at |ϕ| = 0.594, several items showed appreciable cross-loadings across these subcomponents, and one of the emerging factors was predominantly defined by only two items. The three-factor solution therefore provided the clearest balance of simple structure, conceptual coherence, and parsimony. The two-, three-, and four-factor solutions accounted for approximately 42.3%, 47.0%, and 49.9% of extracted variance, respectively. A comparison of the adjacent two-, three-, and four-factor solutions is provided in Supplementary Table S9.

The final three discourse-related factors were labeled D1) socially driven self-judgment (6 items; α = 0.84), D2) warning-triggered self-appraisal (4 items; α = 0.80), and D3) perceived over-pathologization and normative dissent (9 items; α = 0.85). Because factor orientation in EFA is mathematically arbitrary, the factor corresponding to D2 was initially extracted opposite to the substantive scoring direction of its items. D2 was reoriented for presentation; following this reorientation, factor correlations were ϕ = 0.52 between D1 and D2, ϕ = 0.37 between D1 and D3, and ϕ = 0.13 between D2 and D3. The reorientation does not alter the underlying factor solution, observed domain sum scores, or subsequent analyses. Table 2 presents the retained items and primary loadings; the complete pattern matrix, including all cross-loadings, factor correlations, item-removal history, and adjacent alternative solutions are provided in the Supplementary material.

**Table 2:** Discourse-based survey: factor loadings and communalities.

Factor/Item	Loading	*h* ^2^
D1: Socially driven self-judgment		
1. I have often wondered whether my use of social media is excessive because others see it that way.	0.857	0.714
2. When others talk about addiction or dependence on social media, I wonder whether they mean me as well.	0.805	0.590
3. People in my environment have spoken negatively about how often I use social media.	0.550	0.380
4. It bothers me that I almost automatically view my own use of social media critically because so many negative things are said about it from the outside.	0.535	0.568
5. Sometimes I wonder whether I meet the criteria for “social media addiction,” even though I do not really know what these criteria are.	0.447	0.403
6. My assessment of my use of social media is strongly shaped by public opinions and media coverage.	0.391	0.369
D2: Warning-triggered self-appraisal		
7. When I see how much time I spend on social media each day or week (e.g., in screen time statistics), I feel that it is too much.	0.789	0.614
8. When experts warn against using social media, I automatically think about whether my own behavior might also be affected.	0.685	0.606
9. Notifications or warnings from apps about my usage time make me perceive my own use of social media as more problematic than I did before.	0.652	0.447
10. I sometimes wonder whether I use social media too much because there are so many warnings about it.	0.582	0.461
D3: Perceived over-pathologization and normative dissent		
11. I think that my use of social media would be seen less critically in a more digitally oriented community.	0.667	0.411
12. I experience societal or scientific norms about the amount of social media use as too rigid or moralizing.	0.645	0.581
13. The use of social media is often misunderstood as a psychological problem, even though it is not.	0.629	0.383
14. It unsettles or bothers me when psychological technical terms are applied inaccurately to what, for me, is normal media use.	0.628	0.494
15. I feel that society or the media exaggerate the dangers of social media.	0.616	0.396
16. I feel that I am not allowed to simply enjoy my use of social media because it is constantly emphasized how problematic it is supposed to be.	0.592	0.473
17. I imagine that my use of social media would be received more positively if I lived in different cultural environments or communities.	0.576	0.389
18. It bothers me when my everyday use of social media is quickly labeled as “problematic” or “addictive”.	0.571	0.438
19. I keep hearing that my use of social media has negative consequences (e.g., for sleep or real-life friendships), but that does not apply to me.	0.465	0.219

Extraction method: Principal Axis Factoring. Rotation: Oblimin with Kaiser normalization.

Items are numbered sequentially in this table for presentation; Supplementary Table S7 retains the original administered item numbers.

### 3.3 Correlations

As a next step, correlations between BSMAS scores, SM use per week, and all domains were examined (see Table 3). Table 3 reports Pearson correlations among the observed domain sum scores used in the subsequent analyses, rather than correlations among EFA-derived factor scores. BSMAS scores were positively associated with weekly SM use (*r* = 0.28), but more strongly with the functional domain of F1 emotional self-regulatory engagement and escapism (*r* = 0.62). Smaller positive correlations emerged between BSMAS scores and F3 entertainment and stimulation-driven engagement, F4 instrumental and goal-directed use, and F5 social validation and self-presentation (*r*s = 0.26–0.39); F2 global wellbeing benefit showed essentially no association (*r* = 0.05). BSMAS scores were also substantially correlated with the discourse-related domains D1 socially driven self-judgment (*r* = 0.47) and D2 warning-triggered self-appraisal (*r* = 0.55), and modestly with D3 perceived over-pathologization and normative dissent (*r* = 0.21). The highest correlations between functional and discourse-related domains emerged between F2 global wellbeing benefit and D3 perceived over-pathologization and normative dissent (*r* = 0.55), and between F1 emotional self-regulatory engagement and escapism and D1 socially driven self-judgment (*r* = 0.54).

**Table 3:** Descriptive statistics and Pearson correlations among study variables.

Variable	M	SD	1	2	3	4	5	6	7	8	9	10
1. BSMAS	15.46	5.19	–									
2. Weekly SM use (hours)	21.70	12.82	0.28^***^	–								
3. F1: Emotional self-regulatory engagement and escapism	17.98	7.51	0.62^***^	0.26^***^	–							
4. F2: Global wellbeing benefit	6.87	2.96	0.05	0.13^*^	0.22^***^	–						
5. F3: Entertainment and stimulation-driven engagement	21.13	4.88	0.39^***^	0.24^***^	0.46^***^	0.24^***^	–					
6. F4: Instrumental and goal-directed use	20.45	4.74	0.27^***^	0.13^*^	0.29^***^	0.33^***^	0.43^***^	–				
7. F5: Social validation and self-presentation	9.74	4.67	0.26^***^	0.21^***^	0.33^***^	0.40^***^	0.22^***^	0.30^***^	–			
8. D1: Socially driven self-judgment	16.82	7.73	0.47^***^	0.28^***^	0.54^***^	0.22^***^	0.33^***^	0.30^***^	0.28^***^	–		
9. D2: Warning-triggered self-appraisal	18.41	6.06	0.55^***^	0.29^***^	0.42^***^	0.00	0.41^***^	0.30^***^	0.24^***^	0.54^***^	–	
10. D3: Perceived over-pathologization and normative dissent	28.29	10.80	0.21^***^	0.16^**^	0.36^***^	0.55^***^	0.20^***^	0.30^***^	0.42^***^	0.48^***^	0.19^**^	–

*N* = 291. Functional and discourse domain values represent unweighted sum scores of the retained items; EFA-derived factor scores were not used in these analyses. BSMAS = Bergen Social Media Addiction Scale; SM, social media. ^*^p < 0.05. ^**^p < 0.01. ^***^p < 0.001.

### 3.4 Hierarchical regression analysis

To examine the adjusted associations of behavioral, functional, and discourse-related self-appraisal variables with BSMAS scores, we conducted a hierarchical regression with BSMAS scores as the dependent variable (see Table 4).

**Table 4:** Summary of hierarchical regression analysis for BSMAS sum score.

Model/Predictor	*b*	SE	β	95% CI for *b*	*p*	*R^2^*	Adj. *R^2^*	Δ*R^2^*	Δ*F (df)*
**Model 1**						**0.077**	**0.073**	**0.077**	**23.981 (1, 289)**
Weekly SM use	0.112	0.023	0.277	[0.067, 0.157]	<0.001				
**Model 2**						**0.351**	**0.342**	**0.275**	**40.361 (3, 286)**
Weekly SM use	0.040	0.020	0.098	[−0.001, 0.080]	0.053				
D1: Socially driven self-judgment	0.155	0.043	0.231	[0.071, 0.240]	<0.001				
D2: Warning-triggered self appraisal	0.334	0.049	0.390	[0.237, 0.431]	<0.001				
D3: Perceived over-pathologization and normative dissent	0.004	0.026	0.009	[−0.047, 0.056]	0.864				
**Model 3**						**0.498**	**0.482**	**0.147**	**16.419 (5, 281)**
Weekly SM use	0.023	0.018	0.056	[−0.014, 0.059]	0.220				
D1: Socially driven self-judgment	0.047	0.040	0.070	[−0.032, 0.126]	0.242				
D2: Warning-triggered self appraisal	0.234	0.047	0.273	[0.141, 0.327]	<0.001				
D3: Perceived over-pathologization and normative dissent	−0.014	0.028	−0.028	[−0.068, 0.041]	0.623				
F1: Emotional self-regulatory engagement and escapism	0.303	0.038	0.438	[0.228, 0.378]	<0.001				
F2: Global wellbeing benefit	−0.167	0.096	−0.095	[−0.356, 0.023]	0.084				
F3: Entertainment and stimulation-driven engagement	0.046	0.056	0.044	[−0.064, 0.157]	0.409				
F4: Instrumental and goal-directed use	0.041	0.055	0.037	[−0.067, 0.149]	0.455				
F5: Social validation and self-presentation	0.053	0.055	0.047	[−0.056, 0.161]	0.343				

*b*, unstandardized coefficient; *SE*, standard error; β, standardized coefficient; CI, confidence interval. Bold values indicate model-level summary statistics

In Step 1, weekly SM use was significantly associated with BSMAS scores (β = 0.28, *p* < 0.001), explaining 7.7% of the variance (*R*^2^ = 0.077, adjusted *R*^2^ = 0.073), Δ*F*(1, 289) = 23.98, *p* < 0.001. Adding the three discourse-related self-appraisal domains in Step 2 substantially improved the model (*R*^2^ = 0.351, adjusted *R*^2^ = 0.342, Δ*R*^2^ = 0.275), Δ*F*(3, 286) = 40.36, *p* < 0.001. D1 socially driven self-judgment (β = 0.23, *p* < 0.001) and D2 warning-triggered self-appraisal (β = 0.39, *p* < 0.001) were significant predictors, while weekly SM use was no longer statistically significant (β = 0.10, *p* = 0.053).

In Step 3, inclusion of the five functional domains increased explained variance to 49.8% (*R*^2^ = 0.498, adjusted *R*^2^ = 0.482, Δ*R*^2^ = 0.147), Δ*F*(5, 281) = 16.42, *p* < 0.001. Only F1 emotional self-regulatory engagement and escapism (β = 0.44, *p* < 0.001) and D2 warning-triggered self-appraisal (β = 0.27, *p* < 0.001) remained statistically significant in the fully adjusted model. Variance inflation factors ranged from 1.16 to 1.99, and tolerance values ranged from 0.50 to 0.86, indicating no evidence of problematic multicollinearity among the predictors. Regression diagnostics did not indicate pronounced non-linearity. The residual histogram and normal P-P plot showed modest departures from normality, while the standardized residual-vs.-predicted-value plot showed some increase in residual dispersion at higher predicted values but no severe heteroscedastic pattern. One observation had a standardized residual of 3.64. The maximum Cook's distance across observations was 0.052. As a robustness check, the fully adjusted regression was repeated after excluding the observation with the large residual. In the intermediate Step 2 model, weekly SM use crossed the conventional significance threshold after case exclusion (*p* = 0.033, compared with *p* = 0.053 in the primary analysis). However, the substantive conclusions of the fully adjusted model were unchanged: F1 emotional self-regulatory engagement and escapism (β = 0.441 vs. 0.438 in the primary model) and D2 warning-triggered self-appraisal (β = 0.271 vs. 0.273) remained the only significant predictors, while weekly SM use remained non-significant. The observation was therefore retained in the primary analysis.

### 3.5 Sensitivity analyses addressing conceptual overlap with the BSMAS

Please note that complete coefficients, 95% confidence intervals, reported p values, and model-change statistics for all sensitivity analyses are provided in Supplementary Tables S1–S3. To evaluate conceptual overlaps between discourse-related domains and BSMAS scores, we conducted three sensitivity analyses.

First, to evaluate whether associations between the D1 and D3 discourse-related domains and BSMAS scores were attributable to conceptual overlap, we conducted a sensitivity analysis comparing two otherwise identical hierarchical regression models, Models A and B. As in Section 3.4, both models included weekly SM use in Step 1 and the five functional domains in Step 3. However, in Step 2, Model A included the original D1 socially driven self-judgment and D3 perceived over-pathologization and normative dissent scores, whereas Model B included overlap-reduced D1 and D3 scores after removing items that explicitly referred to addiction, excessive use, or negative consequences. Specifically, Table 2 items 1, 2, and 5 were removed from D1 and Table 2 items 18 and 19 from D3. The resulting revised D1 and D3 scores showed internal consistencies of α = 0.697 and α = 0.829, respectively. D2 was not included in either sensitivity model because removal of potentially overlapping items left too few indicators to adequately represent the construct.

In Model A, using the original D1 and D3 scores, adding the two discourse-related domains in Step 2 increased explained variance from 7.7% to 24.8% (Δ*R*^2^ = 0.171, *p* < 0.001). D1 socially driven self-judgment was a significant predictor (β = 0.443, *p* < 0.001), whereas D3 perceived over-pathologization and normative dissent was not (β = −0.028, *p* = 0.629). Weekly SM use remained significant (β = 0.158, *p* = 0.003). Following inclusion of the five functional domains in Step 3, model A explained 45.4% of the variance (Δ*R*^2^ = 0.206, *p* < 0.001). D1 remained a significant unique predictor (β = 0.182, *p* = 0.002), whereas D3 remained non-significant (β = −0.042, *p* = 0.489). F1 emotional self-regulatory engagement and escapism was the strongest predictor (β = 0.459, *p* < 0.001), while F2 global wellbeing benefit showed a smaller negative association (β = −0.155, *p* = 0.006). Weekly SM use was no longer statistically significant (β = 0.086, *p* = 0.068).

In Model B, using the revised D1 and D3 scores, adding the discourse-related domains in Step 2 increased the explained variance from 7.7% to 18.8% (Δ*R*^2^ = 0.112, *p* < 0.001). Revised D1 was a significant predictor (β = 0.349, *p* < 0.001), whereas revised D3 was not significant (β = −0.013, *p* = 0.832); weekly SM use also remained significant (β = 0.207, *p* < 0.001). After including the five functional domains in Step 3, model B explained 44.2% of the variance (Δ*R*^2^ = 0.254, *p* < 0.001). The association for revised D1 was attenuated and no longer statistically significant (β = 0.110, *p* = 0.057), while revised D3 remained non-significant (β = −0.014, *p* = 0.820). Emotional self-regulatory engagement and escapism was the strongest predictor (β = 0.486, *p* < 0.001), while global wellbeing benefit was negatively associated with BSMAS scores (β = −0.171, *p* = 0.003) and entertainment and stimulation-driven engagement showed a smaller positive association (β = 0.108, *p* = 0.049). Weekly SM use also remained significant (β = 0.102, *p* = 0.031).

Overall, removal of discourse items with explicit conceptual overlap in model B attenuated the association between socially driven self-judgment and BSMAS scores. Revised D1 remained significantly associated with BSMAS scores after controlling for weekly SM use but no longer made a statistically significant unique contribution after including the functional domains. The total variance explained by the fully adjusted models was nevertheless similar: 45.4% for Model A using the original discourse scores and 44.2% for Model B using the overlap-reduced scores.

Because removing potentially overlapping D2 items would have left too few indicators to adequately represent the domain, we conducted a second sensitivity analysis from the outcome side. D2 warning-triggered self-appraisal comprises items assessing whether external warnings, usage-time feedback, or expert messages prompt individuals to evaluate their own SM use as excessive or problematic. BSMAS Item 6 was selected because it explicitly assesses a negative consequence of SM use in work or studies and therefore represented the clearest available BSMAS item for testing whether negative evaluative or consequence-related content disproportionately contributed to the D2 association. The BSMAS was recalculated without this item, and the resulting five-item BSMAS showed acceptable internal consistency (α = 0.741). D2 remained substantially correlated with the reduced BSMAS score (*r* = 0.534, *p* < 0.001), compared with *r* = 0.545 for the original six-item score. Repeating the primary hierarchical regression with the reduced BSMAS score produced a highly similar pattern. Weekly SM use explained 6.5% of the variance in Step 1. Addition of the three discourse-related domains increased explained variance to 33.9% (Δ*R*^2^ = 0.273, *p* < 0.001), with D1 socially driven self-judgment (β = 0.229, *p* < 0.001) and D2 warning-triggered self-appraisal (β = 0.382, *p* < 0.001) being significant. After including the five functional domains, the final model explained 48.7% of the variance (adjusted *R*^2^ = 0.470; Δ*R*^2^ = 0.148, *p* < 0.001). As in the primary analysis in Section 3.4, only D2 warning-triggered self-appraisal (β = 0.265, *p* < 0.001) and F1 emotional self-regulatory engagement and escapism (β = 0.434, *p* < 0.001) were significant. Removing Item 6 produced only minimal attenuation of the D2 association. This sensitivity analysis therefore indicates that the association was not dependent on this single negative-consequence item; however, it does not establish conceptual independence between warning-triggered self-appraisal and broader self-evaluations of one's SM use as problematic.

A third sensitivity analysis examined whether the strong association between F1 emotional self-regulatory engagement and escapism and BSMAS scores was partly attributable to overlap with the BSMAS mood-modification item. We recalculated the BSMAS after excluding Item 3, which assesses using SM to forget personal problems. The resulting five-item score retained acceptable internal consistency (α = 0.766). The correlation between F1 and BSMAS scores decreased from *r* = 0.620 for the original six-item scale to *r* = 0.527 for the reduced scale.

The primary hierarchical regression was then repeated using the reduced BSMAS score as the dependent variable. Weekly SM use explained 6.7% of the variance in Step 1. Addition of the three discourse-related domains increased explained variance to 34.2% (Δ*R*^2^ = 0.274, *p* < 0.001). Addition of the five functional domains in Step 3 increased explained variance by a further 8.3%, resulting in a final *R*^2^ = 0.424 (adjusted *R*^2^ = 0.406; Δ*R*^2^ = 0.083, *p* < 0.001). In the final model, F1 remained a significant unique predictor (β = 0.315, *p* < 0.001), although its standardized coefficient was lower than in the primary model (β = 0.438). D2 warning-triggered self-appraisal also remained significant (β = 0.288, *p* < 0.001), while D1 socially driven self-judgment showed a smaller significant association (β = 0.132, *p* = 0.039). The remaining predictors were not statistically significant. Overall, removal of the BSMAS mood-modification item attenuated but did not eliminate the unique association between F1 and BSMAS scores. Table 5 summarizes all findings.

**Table 5:** Summary of sensitivity analyses.

Sensitivity analysis	Outcome/modification	Key result	Final *R*^2^	Adj. *R*^2^	Interpretation
1a. D1/D3 original matched model	Original D1 and D3; D2 excluded	D1 β = 0.182, *p* = 0.002	0.454	0.439	D1 significant after functional domains
1b. D1/D3 overlap-reduced model	Reduced D1 and D3; D2 excluded	Revised D1 β = 0.110, *p* = 0.057	0.442	0.427	D1 attenuated after overlap reduction
2. D2 sensitivity	BSMAS excluding Item 6	D2 β = 0.265, *p* < 0.001	0.487	0.470	D2 essentially unchanged
3. F1 sensitivity	BSMAS excluding Item 3	F1 β = 0.315, *p* < 0.001	0.424	0.406	F1 attenuated but remained significant

## 4 Discussion

This exploratory pilot study examined self-reported addiction-like symptoms related to SM use from two complementary perspectives: the psychological functions served by SM use and individuals' discourse-related self-appraisals of their own use. The findings should not be interpreted as showing that public discourse causes addiction-like symptoms, nor that elevated BSMAS scores are artifacts of public narratives. Rather, they indicate that, within the present sample, BSMAS scores were associated not only with reported time spent on SM but also with coping-oriented motives and with how participants evaluated their own SM use in relation to perceived social judgments, warnings, and other discourse-related cues. In the fully adjusted primary model, the predictors jointly accounted for 49.8% of the variance in BSMAS scores, with emotional self-regulatory engagement and escapism and warning-triggered self-appraisal emerging as the two significant unique predictors.

The present findings also provide initial evidence for interpretable multidimensional structures for the two newly developed instruments. The Functional SM Use Survey yielded five domains, whereas the Discourse-Based Survey yielded three discourse-related self-appraisal domains. Alternative factor solutions and item-reduction analyses supported these structures in the present dataset; however, because the instruments were developed and evaluated in the same sample, the resulting structures should be regarded as provisional rather than established measurement models. Independent replication and subsequent confirmatory testing will be necessary before drawing stronger conclusions about their psychometric structure.

One notable finding concerned the comparatively limited role of reported time spent on SM. Weekly SM use was positively associated with BSMAS scores at the bivariate level (*r* = 0.28) and was significantly associated with BSMAS scores when entered alone. However, its unique association was attenuated after adding the discourse-related domains (β = 0.10, *p* = 0.053) and was no longer statistically significant in the fully adjusted model. This pattern suggests that self-reported duration alone captured relatively little unique variance once differences in functional motives and discourse-related self-appraisals were considered. It should not, however, be interpreted as demonstrating that time spent on SM is unimportant or unrelated to problematic use, particularly because weekly use was assessed by a single retrospective self-report estimate. Rather, the finding is consistent with arguments that frequency or duration of engagement should not be treated as sufficient evidence of behavioral addiction without considering the function, context, and consequences of the behavior (Billieux et al., 2015; Kardefelt-Winther et al., 2017).

Emotional self-regulatory engagement and escapism showed the strongest bivariate association with BSMAS scores (*r* = 0.62) and remained the strongest unique predictor in the fully adjusted primary model (β = 0.438, *p* < 0.001). This is consistent with previous work suggesting that coping-oriented and affect-regulatory motives are particularly relevant to problematic forms of digital-media engagement (Brown et al., 2026; Yildiz and Uslu, 2023). Importantly, however, the conceptual proximity between F1 and the BSMAS mood-modification component required additional scrutiny. When the BSMAS mood-modification item was removed, the F1—BSMAS correlation decreased from *r* = 0.620 to *r* = 0.527 and the fully adjusted F1 coefficient decreased from β = 0.438 to β = 0.315. Thus, the attenuation is consistent with shared affect-regulation content accounting for part of the original association. At the same time, F1 remained significantly associated with the reduced BSMAS score, indicating that the observed relationship was not dependent on that single BSMAS item. These findings therefore support a more cautious interpretation: coping-oriented SM use and self-reported addiction-like symptoms are substantially related in the present data, but the study does not establish that emotion-regulatory use itself represents pathological or addictive behavior.

The discourse-related results require similar caution. At the observed sum-score level, all three domains were positively related, with the strongest association occurring between D1 socially driven self-judgment and D2 warning-triggered self-appraisal (*r* = 0.54). D1 was also moderately related to D3 perceived over-pathologization and normative dissent (*r* = 0.48), whereas the association between D2 and D3 was smaller (*r* = 0.19). These positive associations indicate that the domains are not opposing psychological processes. Instead, individuals may simultaneously be sensitive to external judgments and warnings while also expressing skepticism toward broader pathologizing narratives. The factor structure nevertheless suggests that these forms of self-appraisal were empirically distinguishable within the present sample.

With respect to BSMAS scores, D2 warning-triggered self-appraisal showed the strongest discourse-related bivariate association (*r* = 0.55), followed by D1 socially driven self-judgment (*r* = 0.47), whereas D3 perceived over-pathologization and normative dissent showed a more modest association (*r* = 0.21). In the primary regression, both D1 and D2 showed unique statistical associations after controlling for weekly SM use, but only D2 remained significant once the functional domains were added (β = 0.273, *p* < 0.001). This finding indicates that participants who reported stronger self-appraisal in response to expert warnings, screen-time feedback, and similar cues also tended to endorse more BSMAS symptoms, even after accounting statistically for the other measured domains. It does not establish that warnings or public narratives caused those symptoms. Because D2 itself asks respondents whether warnings and usage feedback affect how they evaluate their own behavior, it is conceptually close to self-perceived problematic use, and the observed association may reflect discourse-related interpretation, greater underlying difficulties with SM use, or some combination of the two.

The sensitivity analysis addressing D2 provides some reassurance that its association with the BSMAS was not driven solely by the BSMAS item assessing negative consequences for work or studies. When that item was excluded, the correlation between D2 and the reduced BSMAS changed only from *r* = 0.545 to *r* = 0.534, and the fully adjusted D2 coefficient changed from β = 0.273 in the primary model to β = 0.265. Nevertheless, this analysis should not be taken as evidence of full discriminant validity between the constructs. Removing a single BSMAS item does not remove the broader conceptual proximity between warning-triggered self-appraisal and respondents' judgments that their own SM use may be excessive or problematic.

The findings for D1 socially driven self-judgment were more sensitive to item content and model specification. In the sensitivity model using the original D1 and D3 scores while omitting D2, D1 remained a significant unique predictor in the fully adjusted model (β = 0.182, *p* = 0.002). After removing three D1 items with particularly explicit references to addiction, excessive use, or negative consequences, however, the corresponding coefficient was reduced to β = 0.110 and was no longer statistically significant (*p* = 0.057) once the functional domains were entered. This attenuation suggests that part of the original D1 association reflects overlapping evaluative content and/or variance shared with the functional domains. It therefore argues against interpreting D1 as an independent discourse effect on BSMAS scores. Instead, socially driven self-judgment appears to be related to addiction-like symptom endorsement, but the extent of its unique contribution depends on how the construct is operationalized. D3 perceived over-pathologization and normative dissent did not make a significant unique contribution in these matched sensitivity models.

Global wellbeing benefit showed a different pattern. It was essentially unrelated to BSMAS scores at the bivariate level (*r* = 0.05) and was not statistically significant in the fully adjusted primary model (β = −0.095, *p* = 0.084). At the same time, it was strongly positively associated with D3 perceived over-pathologization and normative dissent (*r* = 0.55), suggesting that participants who perceived greater overall benefit from SM also tended to express stronger skepticism toward pathologizing or rigid interpretations of SM use. In the overlap-reduced D1/D3 sensitivity model, global wellbeing benefit emerged as a significant inverse predictor of BSMAS scores (β = −0.171, *p* = 0.003). Because this inverse association was not present bivariately and did not reach significance in the primary model, it should not be interpreted as evidence that perceived SM-related wellbeing is protective. Rather, the finding appears model-dependent and may reflect shared variance or suppression among the correlated predictors. Replication would be necessary before assigning substantive meaning to this effect.

Taken together, the findings are compatible with the possibility that self-reported addiction-like symptoms are associated with more than behavioral exposure alone. In this sample, higher BSMAS scores co-occurred particularly with coping-oriented SM use and with stronger warning-triggered self-appraisal. This does not imply that these processes produce elevated BSMAS scores, that BSMAS responses merely reflect cultural narratives, or that clinically meaningful problematic SM use does not occur. Instead, the findings raise a measurement and interpretation question: when people respond to self-report measures of addiction-like symptoms, their responses may be associated both with how SM functions in their lives and with how they evaluate their own behavior. This interpretation is consistent with broader critiques cautioning against inferring pathology solely from high engagement or symptom counts without considering functional impairment and contextual meaning (Billieux et al., 2015; Kardefelt-Winther et al., 2017).

The discourse-related findings are also broadly consistent with the theoretical perspectives outlined in the Introduction. Socially driven self-judgment is conceptually compatible with social-comparison processes, whereas warning-triggered self-appraisal is consistent with the possibility that externally provided evaluative information can become relevant to self-assessment. Conversely, perceived over-pathologization and normative dissent illustrate that individuals do not necessarily adopt socially available labels uncritically. Importantly, the present study did not directly measure exposure to public discourse, experimentally manipulate framing, or assess the actual content of participants' cultural environments. The data therefore support associations among self-reported appraisals, not claims about the causal influence of public discourse.

Future research could test these preliminary findings using independent samples and methods that more clearly separate behavior from self-evaluation. For example, self-reported motives and appraisals could be combined with objectively recorded usage data, ecological momentary assessments, independent indicators of impairment, or experimental manipulations of how digital behavior is framed. Longitudinal designs would also help establish whether coping-oriented use and warning-triggered self-appraisal precede increases in addiction-like symptom endorsement, result from already perceived difficulties with SM use, or develop reciprocally over time. Such work may help clarify when high SM engagement represents ordinary or adaptive use, coping-oriented engagement, or clinically meaningful impairment.

### 4.1 Limitations

Several limitations substantially constrain the interpretation of these findings. First, the study was cross-sectional. Therefore, the temporal direction among functional motives, discourse-related self-appraisals, and BSMAS scores cannot be established. For example, warning-triggered self-appraisal could contribute to perceiving one's use as problematic, individuals already experiencing greater difficulties could become more attentive to warnings and usage feedback, or both processes could occur simultaneously. Similarly, coping-oriented SM use may precede greater symptom endorsement, may develop in response to distress associated with problematic use, or may simply co-occur with it. Causal interpretations are therefore not warranted.

Second, we assessed all principal constructs using self-report measures within the same survey session. Associations may consequently be inflated by shared method variance, overlapping response tendencies, or participants' general evaluative styles. This issue is particularly relevant because both the novel discourse domains and the BSMAS ask participants to evaluate aspects of their own SM behavior. No *post-hoc* statistical procedure can establish the absence of common-method variance. Future replication using multiple methods like objective usage logs, ecological assessment, independent impairment ratings, or temporally separated measurements, would provide a stronger test of whether the observed relationships persist across measurement sources.

Also, the survey sections were presented in a fixed rather than randomized order, with the BSMAS preceding both newly developed instruments and the Discourse-Based Survey, which appeared last; therefore, order, priming, or fatigue effects cannot be excluded. Responding to addiction-related BSMAS items before completing the discourse items could have potentially influenced later questions about addiction-related concepts. Future studies should consider counterbalancing or randomizing instrument order.

The study did not measure public discourse itself, or participants' actual exposure to media or expert messages, and did also not include any questions regarding how often participants encountered specific discourse-related narratives. Instead, participants' own appraisals regarding perceived judgments, warnings, labels, and feedback were assessed. The present findings therefore cannot establish an influence of broader cultural or media discourses. Both novel instruments were developed and tested within the same sample; item selection, factor extraction and regression results may therefore be dependent on sample-specific characteristics. This is especially relevant for F4) instrumental and goal-directed use (α = 0.71), and F5) social validation and self-presentation (α = 0.69).

Several sensitivity analyses reduced conceptual overlap but cannot establish discriminant validity. The overlap-reduced D1 score contained only three items and had lower internal consistency (α = 0.697), meaning that its attenuated association with the BSMAS may reflect reduced construct coverage or reliability as well as reduced conceptual overlap. Likewise, the D2 sensitivity analysis showed that the warning-triggered association persisted after removing one conceptually proximal BSMAS item, but D2 remains inherently close to self-perceived problematic use. Similarly, the F1 sensitivity analysis showed attenuation after removal of the BSMAS mood-modification item, but F1 and the BSMAS remain conceptually related at the broader level of affectively motivated use. These analyses should therefore be viewed as robustness checks rather than demonstrations that the constructs are fully distinct.

Weekly SM use was based on a retrospective self-estimate rather than device-recorded behavior and did not distinguish between platforms, specific activities, or active and passive forms of use. Consequently, the relatively small unique contribution of weekly use should not be generalized to objective or more fine-grained measures of digital engagement. Also, the sample was a convenience sample recruited through Instagram and Facebook, participation was limited to English-language survey materials, and the sample was non-clinical. Recruitment through SM may have preferentially attracted individuals who are relatively engaged with these platforms. The findings therefore cannot be assumed to generalize to the broader population, to individuals with limited SM use, or to people experiencing clinically significant impairment. In addition, no clinical interviews, independent assessments of impairment, behavioral measures, or longitudinal follow-up were conducted. Accordingly, BSMAS scores in this study should be interpreted as self-reported addiction-like symptoms rather than evidence of a diagnosed addictive disorder.

Despite these limitations, the present exploratory findings suggest that variation in BSMAS scores may be associated with both the functions that SM use serves and users' discourse-related self-appraisals. The results do not demonstrate that addiction-like symptom reports are produced by cultural narratives or that elevated BSMAS scores lack clinical relevance. Rather, they support further investigation of whether functional motives, perceived impairment, behavioral indicators, and the interpretive context in which individuals evaluate their own SM use are jointly associated with self-reported problematic-use measures. Such work may provide a more differentiated understanding of high and potentially problematic SM engagement without presuming either that frequent use is inherently pathological or that reported difficulties are merely products of discourse.

## Data Availability

The datasets presented in this article are not readily available because consent obtained from participants did not include permission for public data sharing, redistribution, or deposition in a public repository. Requests to access the datasets should be directed to mario.lehenbauer@sfu.ac.at.
