# Supplementary material for: Beyond time spent online: exploring functional motives, discourse-related self-appraisals, and social media addiction-like symptoms
Source: Front Psychol. 2026 Oct 27;17:1875894. doi: 10.3389/fpsyg.2026.1875894 (PMC13747956; doi:10.3389/fpsyg.2026.1875894)
Supplement: Supplementary file 1 [file Data_Sheet_1.pdf]

Supplementary Material

Overview

Supplementary Table	Content	Page
S1: Matched sensitivity analyses examining conceptual overlap between D1/D3 discourse-related domains and BSMAS scores	Panel A: Original D1 and D3 scores<br>Panel B: Overlap-reduced D1 and D3 scores	2
S2: Sensitivity analysis for D2 warning-triggered self-appraisal	Regression using the 5-item BSMAS excluding Item 6	4
S3: Sensitivity analysis for F1 mood-modification overlap	Regression using the 5-item BSMAS excluding Item 3	5
S4: Final Functional SM Use Survey EFA	Final 18-item, five-factor pattern matrix, with all factor loadings/cross-loadings, communalities; additional panel with factor-correlation matrix.	6
S5: Functional Survey item-removal sequence	Covers process from 31 to final 18 items, including each removed administered item, iteration at which removed, relevant loading(s), communality where useful, and concise statistical/conceptual rationale.	8
S6: Functional factor-retention evidence	Comparison of 4-, 5-, and 6-factor solutions using the final 18-item set, including variance explained and concise structural reason for rejecting 4 and 6 factors.	10
S7: Final Discourse-Based Survey EFA	Final 19-item, three-factor full pattern matrix, all cross-loadings, communalities, and factor-correlation matrix.	11
S8: Discourse Survey item-removal sequence	Covers process from 27 to final 19 items, including removed administered items, iteration, loading/cross-loading/communality evidence, and concise rationale.	13
S9: Discourse factor-retention evidence	Comparison of 2-, 3-, and 4-factor solutions on the final 19 items, including variance explained and concise structural reason for rejecting 2 and 4 factors.	14

Supplementary Table S1.

Matched sensitivity analyses examining conceptual overlap between D1/D3 discourse-related domains and BSMAS scores

Panel A: Original D1 and D3 scores

Model / Predictor	*b*	SE	β	95% CI for *b*	*p*	R^2	Adj. R^2	ΔR^2	ΔF (*df*)
**Model 1**						**.077**	**.073**	**.077**	**23.981 (1, 289)**
Weekly SM use	.112	.023	.277	[.067, .157]	< .001				
**Model 2**						**.248**	**.240**	**.171**	**32.667 (2, 287)**
Weekly SM Use	.064	.022	.158	[.022, .107]	.003				
D1: Socially Driven Self-Judgment	.298	.040	.443	[.218, .377]	< .001				
D3: Perceived Over-Pathologization and Normative Dissent	-.014	.028	-.028	[-.069, .042]	.629				
**Model 3**						**.454**	**.439**	**.206**	**21.332 (5, 282)**
Weekly SM Use	.035	.019	.086	[-.003, .072]	.068				
D1: Socially Driven Self-Judgment	.123	.039	.182	[.047, .198]	.002				
D3: Perceived Over-Pathologization and Normative Dissent	-.020	.029	-.042	[-.077, .037]	.489				
F1: Emotional Self-Regulatory Engagement and Escapism	.317	.039	.459	[.240, .395]	< .001				
F2: Global Wellbeing Benefit	-.271	.098	-.155	[-.463, -.079]	.006				
F3: Entertainment and Stimulation-Driven Engagement	.104	.057	.097	[-.009, .216]	.071				
F4: Instrumental and Goal-Directed Use	.075	.057	.069	[-.036, .187]	.184				
F5: Social Validation and Self-Presentation	.083	.057	.075	[-.030, .196]	.148				

Note. b = unstandardized coefficient; SE = standard error; β = standardized coefficient; CI = confidence interval.

Supplementary Table S1 continued

Panel B: Overlap-reduced D1 and D3 scores

Model / Predictor	*b*	SE	β	95% CI for *b*	*p*	R^2	Adj. R^2	ΔR^2	ΔF *(df)*
**Model 1**						**.077**	**.073**	**.077**	**23.981 (1, 289)**
Weekly SM use	.112	.023	.277	[.067, .157]	< .001				
**Model 2**						**.188**	**.180**	**.112**	**19.731 (2, 287)**
Weekly SM Use	.084	.022	.207	[.040, .127]	< .001				
Overlap-reduced D1: Socially Driven Self-Judgment	.470	.086	.349	[.300, .640]	< .001				
Overlap-reduced D3: Perceived Over-Pathologization and Normative Dissent	-.008	.038	-.013	[-.084, .067]	.832				
**Model 3**						**.442**	**.427**	**.254**	**25.696 (5, 282)**
Weekly SM Use	.041	.019	.102	[.004, .079]	.031				
Overlap-reduced D1: Socially Driven Self-Judgment	.149	.078	.110	[-.005, .302]	.057				
Overlap-reduced D3: Perceived Over-Pathologization and Normative Dissent	-.009	.038	-.014	[-.084, .066]	.820				
F1: Emotional Self-Regulatory Engagement and Escapism	.336	.039	.486	[.259, .414]	< .001				
F2: Global Wellbeing Benefit	-.300	.099	-.171	[-.495, -.105]	.003				
F3: Entertainment and Stimulation-Driven Engagement	.114	.058	.108	[.001, .228]	.049				
F4: Instrumental and Goal-Directed Use	.092	.057	.084	[-.020, .204]	.106				
F5: Social Validation and Self-Presentation	.073	.058	.066	[-.042, .187]	.211				

Note. b = unstandardized coefficient; SE = standard error; β = standardized coefficient; CI = confidence interval.

Supplementary Table S2.

Sensitivity analysis predicting BSMAS scores after removal of Item 6 (negative-impact item)

Model / Predictor	*b*	SE	β	95% CI for *b*	*p*	R^2	Adj. R^2	ΔR^2	ΔF (*df*)
**Model 1**						**.065**	**.062**	**.065**	**20.209 (1, 289)**
Weekly SM use	.088	.020	.256	[.049, .126]	< .001				
**Model 2**						**.339**	**.330**	**.273**	**39.437 (3, 286)**
Weekly SM Use	.026	.017	.076	[-.008, .060]	.136				
D1: Socially Driven Self-Judgment	.130	.037	.229	[.058, .202]	< .001				
D2: Warning-Triggered Self Appraisal	.277	.042	.382	[.194, .360]	< .001				
D3: Perceived Over-Pathologization and Normative Dissent	.014	.022	.033	[-.030, .058]	.546				
**Model 3**						**.487**	**.470**	**.148**	**16.165 (5, 281)**
Weekly SM Use	.011	.016	.031	[-.020, .042]	.501				
D1: Socially Driven Self-Judgment	.039	.034	.068	[-.029, .106]	.258				
D2: Warning-Triggered Self Appraisal	.192	.041	.265	[.112, .272]	< .001				
D3: Perceived Over-Pathologization and Normative Dissent	-.011	.024	-.027	[-.058, .036]	.648				
F1: Emotional Self-Regulatory Engagement and Escapism	.254	.033	.434	[.190, .318]	< .001				
F2: Global Wellbeing Benefit	-.105	.082	-.071	[-.267, .057]	.202				
F3: Entertainment and Stimulation-Driven Engagement	.036	.048	.040	[-.059, .130]	.459				
F4: Instrumental and Goal-Directed Use	.050	.047	.054	[-.043, .142]	.290				
F5: Social Validation and Self-Presentation	.059	.047	.063	[-.034, .152]	.213				

Note. The dependent variable was a five-item BSMAS score excluding Item 6, which assesses whether SM use had a negative impact on work or studies. The reduced BSMAS showed α = .741. All three original discourse-related domain scores and all five functional-domain scores were retained

b = unstandardized coefficient; SE = standard error; β = standardized coefficient; CI = confidence interval.

All ΔF tests were significant at $p < .001$.

Supplementary Table S3.

Sensitivity analysis predicting BSMAS scores after removal of Item 3 (mood-modification item)

Model / Predictor	*b*	SE	β	95% CI for *b*	p	R^2	Adj. R^2	ΔR^2	ΔF (*df*)
**Model 1**						**.067**	**.064**	**.067**	**20.898 (1, 289)**
Weekly SM use	.089	.019	.260	[.050, .127]	< .001				
**Model 2**						**.342**	**.332**	**.274**	**39.689 (3, 286)**
Weekly SM Use	.028	.017	.083	[-.006, .062]	.103				
D1: Socially Driven Self-Judgment	.142	.036	.251	[.070, .214]	< .001				
D2: Warning-Triggered Self Appraisal	.279	.042	.386	[.196, .361]	< .001				
D3: Perceived Over-Pathologization and Normative Dissent	-.013	.022	-.031	[-.056, .031]	.570				
**Model 3**						**.424**	**.406**	**.083**	**8.079 (5, 281)**
Weekly SM Use	.019	.017	.054	[-.014, .051]	.267				
D1: Socially Driven Self-Judgment	.075	.036	.132	[.004, .146]	.039				
D2: Warning-Triggered Self Appraisal	.208	.043	.288	[.124, .292]	< .001				
D3: Perceived Over-Pathologization and Normative Dissent	-.017	.025	-.041	[-.066, .033]	.506				
F1: Emotional Self-Regulatory Engagement and Escapism	.184	.034	.315	[.116, .251]	< .001				
F2: Global Wellbeing Benefit	-.166	.087	-.112	[-.336, .005]	.058				
F3: Entertainment and Stimulation-Driven Engagement	.037	.051	.041	[-.063, .137]	.468				
F4: Instrumental and Goal-Directed Use	.042	.049	.045	[-.055, .139]	.398				
F5: Social Validation and Self-Presentation	.039	.050	.041	[-.060, .137]	.438				

Note. The dependent variable was a five-item BSMAS score excluding Item 3, which assesses using SM to forget about personal problems. The reduced BSMAS showed α = .766. All original discourse-related and functional-domain scores were retained.
b = unstandardized coefficient; SE = standard error; β = standardized coefficient; CI = confidence interval.
All ΔF tests were significant at $p < .001$.

Supplementary Table S4.

Final five-factor exploratory factor solution for the 18-item Functional SM Use Survey

Panel A. Pattern matrix and extraction communalities

Item	Item content	F1	F2	F3	F4	F5	h^2
**F1: Emotional Self-Regulatory Engagement and Escapism**							
12	I use social media to "escape" from stressful situations for a moment.	**.806**	-.168	.074	.053	.025	.728
15	When I feel stressed, anxious, or overwhelmed, I use social media to distract myself.	**.805**	-.164	.122	.103	-.039	.764
8	Social media helps me cope better with difficult emotions.	**.741**	.180	-.061	-.018	.034	.600
2	I use social media to calm myself down internally after exhausting or emotionally stressful situations.	**.709**	.018	.136	.031	-.062	.584
25	I use social media to have the feeling that I have my emotions better under control.	**.571**	.154	-.125	-.045	.170	.429
**F2: Global Wellbeing Benefit**							
20	I feel that using social media supports my overall well-being.	.077	**.795**	.164	.073	-.019	.725
27	I feel that social media has a positive impact on my daily life.	-.036	**.719**	.036	.115	.133	.658
**F3: Entertainment and Stimulation-Driven Engagement**							
14	I use social media because certain content strongly appeals to and stimulates me.	-.036	.103	**.928**	-.044	.028	.826
13	I use social media because I find the content entertaining.	-.035	.043	**.671**	.073	-.025	.477
23	I use social media because some content is especially stimulating for me.	.075	.147	**.648**	.066	.124	.606
5	I use social media to combat boredom.	.238	-.138	**.492**	-.012	-.042	.384
**F4: Instrumental and Goal-Directed Use**							
22	I use social media to get inspiration and ideas (e.g., for fashion, home decor, work, hobbies, new recipes, etc.).	.020	-.026	.080	**.693**	.063	.564
17	I use social media to get information about topics that are important for me personally, for my job, or for my education.	.001	.063	-.050	**.548**	-.107	.274
30	I use social media to find out what is new online.	-.029	-.091	.208	**.528**	.188	.479
31	I use social media to discover new hobbies or interests.	.088	.099	-.027	**.521**	.112	.375
**F5: Social Validation and Self-Presentation**							
10	I use social media because positive feedback or reactions (e.g., likes, matches, messages) strengthen my self-esteem.	.038	.021	.047	-.063	**.836**	.725

Item	Item content	F1	F2	F3	F4	F5	h^2
28	I use social media to present myself (e.g., through photos, posts, or stories).	-.002	-.092	.029	.160	**.658**	.478
18	I use social media to meet new people (e.g., through dating apps, groups, or communities).	.039	.134	-.031	-.049	**.443**	.259

Panel B. Factor Correlation Matrix

Factor	F1	F2	F3	F4	F5
F1	-				
F2	.131	-			
F3	.388	.023	-		
F4	.223	.200	.439	-	
F5	.338	.387	.164	.260	-

Note. Extraction method = principal axis factoring; rotation method = direct Oblimin with Kaiser normalization. All pattern coefficients are shown without suppression. Bold values indicate the primary loading used to define each factor. h^2 = extraction communality. F1 = Emotional Self-Regulatory Engagement and Escapism; F2 = Global Wellbeing Benefit; F3 = Entertainment and Stimulation-Driven Engagement; F4 = Instrumental and Goal-Directed Use; F5 = Social Validation and Self-Presentation. The F5 factor was extracted by SPSS in the opposite arbitrary direction to the substantive scoring of its items. For consistency with the manuscript, F5 was reoriented for presentation by multiplying all F5 pattern coefficients and factor correlations involving F5 by -1. This sign reversal does not change the factor solution, communalities, observed domain scores, or subsequent analyses.

Supplementary Table S5.

Item-Removal Sequence for the Functional SM Use Survey

EFA Iteration	Removed item, Item wording	Relevant EFA evidence	Rationale
1: 31 items	1. I use social media to get support from other users.	Largest $|\lambda|$ = .294; h^2= .214	Weak factor representation and low communality.
1: 31 items	3. I use social media to relax.	$|\lambda|$ = .315 and .494; h^2 = .425	Substantial cross-loading and ambiguous factor membership.
1: 31 items	11. Using social media gives me a sense of motivation and inspiration.	Largest $|\lambda|$ = .278; h^2= .367	No salient primary loading and diffuse factor representation.
1: 31 items	29. I use social media to feel internally stimulated or excited.	$|\lambda|$ = .439 and .368; h^2 = .540	Substantial cross-loading and insufficiently clear factor membership.
2: 27 items	4. I use social media to stay in touch with other people.	Primary $|\lambda|$ = .335; secondary $|\lambda|$ = .247; h^2 = .246	Weak primary loading, low communality, and limited specificity relative to the emerging domains.
2: 27 items	6. Out of habit, I check social media several times a day.	Primary $|\lambda|$ = .465; h^2= .337	Statistically moderate representation, but content reflects habitual checking rather than a distinct functional motive; removed to improve conceptual coherence.
2: 27 items	21. I use social media to pass the time while waiting (e.g., when waiting for the bus or train).	$|\lambda|$ = .387 and .358; h^2 = .397	Clear cross-loading and ambiguous factor membership.
3: 24 items	9. When I use social media, I usually have a clear goal in mind (e.g., specific tasks such as searching for information, sending messages, posting something, etc.).	$|\lambda|$ = .416 and .326; h^2 = .266	Cross-loading combined with low communality.
4: 23 items	7. Social media helps me meet certain personal or psychological needs that would otherwise remain unmet.	Primary $|\lambda|$ = .494; secondary $|\lambda|$ = .214; h^2 = .470	Acceptable statistical performance but conceptually broad and nonspecific; removed to improve domain specificity and interpretability.
5: 22 items	19. I deliberately limit my use of social media when I have other important things to do.	Primary $|\lambda|$ = .291; h^2= .086	Very weak factor representation and communality; content also concerns regulation/limitation of use rather than a function motivating SM use.

EFA Iteration	Removed item, Item wording	Relevant EFA evidence	Rationale
6: 21 items	24. I use social media to experience intense feelings or inner tension.	Primary |λ| = .491; h^2= .397	Moderate loading, but content was conceptually inconsistent with the emerging coping, calming, and escapism domain.
6: 21 items	26. Using social media gives me a sense of structure or routine in my everyday life.	Primary |λ| = .518; secondary |λ| = .214; h^2 = .357	Acceptable primary loading but content reflected routine/structure rather than the affect-regulatory construct characterizing the emerging factor.
7: 19 items	16. I use social media because it makes me feel that I am not alone with my problems.	Primary |λ| = .593; secondary |λ| = .296; h^2 = .537	Statistically viable, but content reflected social reassurance/connectedness rather than intrapersonal emotion regulation and escapism; it also had the lowest primary loading within that cluster and a near-threshold secondary loading.
8: 18 items	—	Final solution	No further items removed.

Note. λ = pattern loading; h^2 = extraction communality. Absolute loading values are reported because factor sign is arbitrary. Item-removal decisions considered statistical performance and conceptual interpretability jointly rather than applying numerical cutoffs as automatic deletion rules.

Supplementary Table S6.

Comparison of Alternative Factor Solutions for the Final 18-Item Functional SM Use Survey

Factor solution	Cumulative extracted variance (%)	Key structural evidence	Interpretation
**4 factors**	**50.3**	The two global-wellbeing items loaded on the same factor as social-validation/self-presentation content: Item 20 = .690 and Item 27 = .775, together with Item 10 = .511, Item 18 = .430, and Item 28 = .326. Item 28 also showed a loading of .294 on another factor.	**Under-extraction.** Global wellbeing was not differentiated from social-validation/self-presentation content, producing a broader and conceptually heterogeneous factor.
**5 factors**	**55.2**	The final solution separated emotional self-regulation/escapism, global wellbeing, entertainment/stimulation, instrumental/goal-directed use, and social validation/self-presentation. Primary loadings ranged from .443 to .928, with no secondary loading of $\lvert\lambda\rvert \geq .30$.	**Retained solution.** Provided the clearest simple structure and the most conceptually coherent differentiation of the five functional domains.
**6 factors***	**-**	The initial forced six-factor PAF did not converge within 25 extraction iterations. When rerun with the maximum increased to 100 iterations, extraction was terminated at iteration 100 because the communality of one variable exceeded 1.0.	**No admissible completed six-factor solution.** The six-factor extraction was therefore not interpreted.

Note. All factor analyses used the same final 18-item pool and principal axis factoring with direct Oblimin rotation and Kaiser normalization. Absolute loading values are reported where factor sign is arbitrary. The initial eigenvalue sequence for the 18-item correlation matrix supported five factors: 5.597, 2.159, 1.972, 1.207, and 1.124, followed by a sixth eigenvalue of .749. Factor retention was based jointly on eigenvalues, scree-plot inspection, simple structure, and conceptual interpretability rather than on variance explained alone. The complete retained five-factor pattern matrix and factor-correlation matrix are reported in Supplementary Table S4.

*The forced six-factor solution did not yield an admissible completed solution. The initial analysis did not converge within the default maximum of 25 extraction iterations. When the analysis was repeated with the maximum increased to 100 iterations, the communality of one variable exceeded 1.0 at iteration 100 and extraction was terminated. Accordingly, statistics from the unfinished six-factor solution are not interpreted.

Supplementary Table S7.
Final three-factor exploratory factor solution for the 19-Item Discourse-Based Survey

Panel A. Pattern Matrix and Extraction Communalities

Item	Item content	D1	D2	D3	h^2
**D1: Socially Driven Self-Judgment**					
24	I have often wondered whether my use of social media is excessive because others see it that way.	**.857**	.011	-.053	.714
23	When others talk about addiction or dependence on social media, I wonder whether they mean me as well.	**.805**	-.003	-.115	.590
27	People in my environment have spoken negatively about how often I use social media.	**.550**	.035	.107	.380
25	It bothers me that I almost automatically view my own use of social media critically because so many negative things are said about it from the outside.	**.535**	.108	.293	.568
16	Sometimes I wonder whether I meet the criteria for “social media addiction,” even though I do not really know what these criteria are.	**.447**	.226	.096	.403
14	My assessment of my use of social media is strongly shaped by public opinions and media coverage.	**.391**	.075	.298	.369
**D2: Warning-Triggered Self-Appraisal**					
5	When I see how much time I spend on social media each day or week (e.g., in screen time statistics), I feel that it is too much.	.000	**.789**	-.065	.614
6	When experts warn against using social media, I automatically think about whether my own behavior might also be affected.	.182	**.685**	-.164	.606
7	Notifications or warnings from apps about my usage time make me perceive my own use of social media as more problematic than I did before.	-.016	**.652**	.116	.447
3	I sometimes wonder whether I use social media too much because there are so many warnings about it.	.138	**.582**	.064	.461
**D3: Perceived Over-Pathologization and Normative Dissent**					
12	I think that my use of social media would be seen less critically in a more digitally oriented community.	-.173	.149	**.667**	.411
18	I experience societal or scientific norms about the amount of social media use as too rigid or moralizing.	.229	.004	**.645**	.581
10	The use of social media is often misunderstood as a psychological problem, even though it is not.	-.020	-.111	**.629**	.383
21	It unsettles or bothers me when psychological technical terms are applied inaccurately to what, for me, is normal media use.	.101	.112	**.628**	.494
2	I feel that society or the media exaggerate the dangers of social media.	.010	-.228	**.616**	.396

Item	Item content	D1	D2	D3	h^2
11	I feel that I am not allowed to simply enjoy my use of social media because it is constantly emphasized how problematic it is supposed to be.	.164	.060	**.592**	.473
22	I imagine that my use of social media would be received more positively if I lived in different cultural environments or communities.	.040	.122	**.576**	.389
9	It bothers me when my everyday use of social media is quickly labelled as “problematic” or “addictive.”	.058	.203	**.571**	.438
15	I keep hearing that my use of social media has negative consequences (e.g., for sleep or real-life friendships), but that does not apply to me.	.010	-.126	**.465**	.219

Panel B. Factor Correlation Matrix

Factor	D1	D2	D3
D1	-		
D2	.518	-	
D3	.371	.131	-

Note. Extraction method = principal axis factoring; rotation method = direct Oblimin with Kaiser normalization. All pattern coefficients are shown without suppression. Bold values indicate the primary loading used to define each factor. h^2 = extraction communality. D1 = Socially Driven Self-Judgment; D2 = Warning-Triggered Self-Appraisal; D3 = Perceived Over-Pathologization and Normative Dissent. Because factor orientation in EFA is mathematically arbitrary, D2 was initially extracted in the opposite direction to the substantive scoring of its items. For consistency with the manuscript, D2 was reoriented for presentation by multiplying all D2 pattern coefficients and factor correlations involving D2 by -1. This sign reversal does not change the factor solution, communalities, observed domain scores, or subsequent analyses.

Supplementary Table S8.

Item-Removal Sequence for the Discourse-Based Survey

EFA Iteration	Removed item, Item wording	Relevant EFA evidence	Rationale
1: 27 items	1. Although I use social media a lot, I only started thinking about it after I heard or read critical opinions, articles, or studies about it.	Largest $\lvert\lambda\rvert$ = .284; h^2 = .159	No salient primary loading and very low communality.
1: 27 items	4. I experience my use of social media as mostly positive, but I still wonder whether it is “too much” because others say so.	$\lvert\lambda\rvert$ = .416 and .339; h^2 = .312	Clear cross-loading and ambiguous factor membership.
1: 27 items	8. I find it stressful that, when I use social media, I constantly have in the back of my mind that others might see it as “too much” or “unhealthy.”	Primary $\lvert\lambda\rvert$ = .385; secondary $\lvert\lambda\rvert$ = .286; h^2 = .391	Modest primary loading with near-threshold secondary loading; content also combined social judgment with emotional distress, reducing simple structure.
1: 27 items	13. I often feel that I am not using my time meaningfully when I am online on social media because that is often said by others.	Primary $\lvert\lambda\rvert$ = .336; secondary $\lvert\lambda\rvert$ = .289; h^2 = .355	Weak primary loading with an almost equally strong secondary loading, indicating ambiguous factor representation.
1: 27 items	26. Sometimes I worry that my use of social media in the presence of other people makes me less attentive to them.	Primary $\lvert\lambda\rvert$ = .386; h^2 = .236	Modest loading and low communality; content primarily reflects a perceived interpersonal consequence of SM use rather than discourse-related self-appraisal.
2: 22 items	17. I feel under pressure when I use social media more often than my friends or family.	$\lvert\lambda\rvert$ = .331, .260, and .254; h^2 = .424	Diffuse representation across all three factors with only a weak primary loading; poor simple structure.
2: 22 items	19. In some professional or social circles, I feel that my use of social media is judged more negatively than in others.	Primary $\lvert\lambda\rvert$ = .333; h^2 = .270	Weak primary loading and low communality.
2: 22 items	20. I feel under pressure to reduce my use of social media because that is seen as healthy.	$\lvert\lambda\rvert$ = .383 and .363; h^2 = .477	Nearly equivalent loadings on two factors, indicating substantial cross-loading and unclear factor membership.
3: 19 items	-	Final solution	No further items removed.

Note. λ = pattern loading; h² = extraction communality. Absolute loading values are reported because factor sign is arbitrary. Item-removal decisions considered statistical performance, simple structure, and conceptual interpretability jointly rather than applying numerical cutoffs as automatic deletion rules.

Supplementary Table S9.

Comparison of Alternative Factor Solutions for the Final 19-Item Discourse-Based Survey

Factor solution	Cumulative extracted variance (%)	Key structural evidence	Interpretation
**2 factors**	**42.3**	Warning-triggered items and socially driven self-judgment items loaded together on one broad factor. For example, warning items 3, 5, 6, and 7 loaded at |λ| = .650, .706, .782, and .580, respectively, while socially driven self-judgment items 16, 23, 24, 25, and 27 loaded at |λ| = .575, .624, .676, .534, and .475. Item 14 also showed similar loadings across the two factors (.387 and .363).	**Under-extraction.** The solution failed to distinguish socially driven self-judgment from warning-triggered self-appraisal.
**3 factors**	**47.0**	The retained solution separated socially driven self-judgment, warning-triggered self-appraisal, and perceived over-pathologization/normative dissent. Absolute primary loadings ranged from .391 to .857, with no secondary loading reaching |.30|.	**Retained solution.**Provided the clearest simple structure and the most conceptually coherent differentiation of the three discourse-related domains.
**4 factors**	**49.9**	Perceived over-pathologization and normative dissent split across two strongly related subcomponents (|φ| = .594). One emerging factor was predominantly defined by Items 22 (|λ| = .810) and 12 (|λ| = .478), while several D3 items showed appreciable loadings across the two subcomponents, including Item 2 (.254/.394), Item 18 (.261/.429), and Item 21 (.333/.356).	**Over-extraction.** The additional factor fragmented the normative-dissent/over-pathologization domain without producing a clearly independent construct.

Note. All solutions used the same final 19-item pool and principal axis factoring with direct Oblimin rotation and Kaiser normalization. Absolute loading values are reported where factor sign is arbitrary. The initial eigenvalues for the 19-item correlation matrix were 6.266, 2.897, 1.258, and .933 for the first four factors. Factor retention was based jointly on eigenvalues, scree-plot inspection, simple structure, and conceptual interpretability rather than on variance explained alone. The complete retained three-factor pattern matrix and factor-correlation matrix are reported in Supplementary Table S7.
